# Effect of Garlic (*Allium sativum*) Supplementation on Premenstrual Disorders: A Randomized, Double-Blind, Placebo-Controlled Trial

**DOI:** 10.1155/2021/9965064

**Published:** 2021-11-01

**Authors:** Fatemeh Jafari, Malihe Tabarrai, Alireza Abbassian, Farhad Jafari, Mohammad Hossein Ayati

**Affiliations:** ^1^Department of Traditional Medicine, School of Persian Medicine, Tehran University of Medical Sciences, Tehran, Iran; ^2^Social Medicine, Department of Health and Community Medicine, School of Medicine, Shahed University, Tehran, Iran

## Abstract

**Background:**

Premenstrual disorders involve physical, behavioral, and mood variations that affect women of childbearing age and interfere with family relationships, household responsibilities, professional duties, and social activities.

**Objectives:**

Considering the side effects of conventional medications, their use is not recommended except in severe cases of premenstrual disorders. Nowadays, there is a tendency to use traditional and complementary medicine that offers various treatments. The purpose of the current study was to investigate the impacts of garlic as a herbal medicine on the severity of premenstrual symptoms.

**Methods:**

This study was a double-blind, randomized, controlled trial. After identification of participants with moderate-to-severe PMS through the premenstrual symptoms screening tools questionnaire (PSST), they were randomly assigned to placebo (*n* = 64) or garlic (*n* = 65) groups. Each participant received one tablet daily for three consecutive cycles and logged the severity of their symptoms in the PSST questionnaire during the intervention period.

**Results:**

There was no significant difference between the two groups in the baseline level of premenstrual symptoms before the intervention. After treatment with garlic for three consecutive cycles, the total score of the severity of premenstrual symptoms significantly (*P* < 0.001) reduced from 34.09 ± 7.31 to 11.21 ± 7.17. In the placebo group, this score changed from 33.35 ± 7.96 to 24.28 ± 7.22. The difference between mean changes in the two groups was 13.78, with a 95% Confidence Interval (CI) of 11.23–16.33. No serious side effects were observed in either group.

**Conclusion:**

Our findings highlight the potential effect of garlic in reducing the severity of premenstrual symptoms; therefore, the use of garlic can be considered as an alternative therapy in the prevention and treatment of premenstrual disorders.

## 1. Introduction

Premenstrual disorder (PMD) involves affective, behavioral, and somatic symptoms which occur monthly during the luteal phase of the menstrual cycle and subside after the onset of menstruation [1, 2]. The International Society for the Study of Premenstrual Disorders (ISPMD) newly issued diagnostic standards for PMD present both premenstrual syndrome (PMS) and premenstrual dysphoric disorder (PMDD) as one disorder called PMD. Overall, 20 to 30% of women experience clinically significant PMS symptoms and 3–8% experience symptoms meeting PMDD criteria delineated by the Diagnostic and Statistical Manual of Mental Disorders V (DSM-V) [3]. PMDD can disrupt daily activities at home, in the workplace, and during social interactions, and the symptoms can range from moderate to severe in intensity [4–6]. Daily prospective charting of two menstrual cycles to verify the timing of the symptoms precisely is considered essential by all published standards. An asymptomatic week early in the follicular phase is needed for a definitive diagnosis of PMD and to distinguish psychiatric disorders from it [2, 7].

However, the exact etiology of PMDD is still unknown; it seems that instabilities in gonadal sex steroids, predominantly progesterone, play a role in the pathogenesis of PMDD [8–10]. The metabolites of progesterone with neuroactive properties, including allopregnanolone (3a-hydroxy-5a-pregnan-20-one) and pregnanolone (3a-hydroxy-5b-pregnane-20-one), are positive modulators of gamma-aminobutyric acid (GABA), which is the primary inhibitory neurotransmitter in the brain. Altered functional sensitivity of the GABA receptor and decreased serotonin activity in women with PMD have been reported [11–13]. Consumption of high-calorie diets, sugar, and fat has been identified as crucial risk factor for PMS [14]. On the other hand, enough intake of vitamin D and calcium [15, 16], magnesium, vitamin B1, and vitamin B6 [16–19] might also be beneficial.

Non-pharmacological interventions, such as aerobic exercise, decreased caffeine intake, and increased calcium and carbohydrate intake (before menstruation), are useful for alleviating PMS, but do not improve PMDD symptoms [20, 21]. Some medications, including fluoxetine, sertraline, paroxetine, and oral contraceptives, have been approved by Food and Drug Administration (FDA) in severe PMD [22, 23]. Finally, surgical menopause can be used as the last option to suppress hormones in PMDD [1].

Nowadays, because of the side effects of pharmacological interventions, people are becoming more inclined to use complementary and alternative medicine (CAM) for PMD [24]. Persian medicine as a modality of CAM introduces suitable approaches; for example, Ibn-e-Sina (Avicenna) in his book “The Canon of Medicine” suggested any changes in the menstrual pattern in terms of quantity, quality, and the onset of menstruation could lead to premenstrual disorders. Symptoms potentially subside with regular, adequate, and moderate menstruation [25]. For this, exercise, diet, herbal remedies, and wet cupping are recommended [26].

Among the different modalities of CAM, herbal medicine is the most popular method [27]. Some herbs such as vitex agnus castus (VAC) or chasteberry and *Hypericum perforatum* (St. John's wort) can be effective in the control of PMD symptoms, but it is essential to perform these treatments after gathering sufficient evidence [28–30]. Garlic (*Allium sativum*) is an herb with immunoregulatory effects [31] and reduces anxiety and depression behaviors in diabetic rats, possibly by reducing brain oxidative stress [32]. Recent animal studies have found evidence of the effects of garlic on reducing cognitive and behavioral symptoms through interference with neurotransmitters [33–35]. According to Persian medicine references, garlic is suggested as one of the herbal medicines that can be effective in PMS through lowering blood viscosity and menstruation regulation.

Although research has investigated the effect of garlic on other female disorders such as dysmenorrhea and PCOS, there is a paucity of studies assessed the effects of garlic on PMS. Therefore, the present study aims to explore the efficacy of garlic on young women with PMS.

## 2. Materials and Methods

### 2.1. Study Design and Setting

The current study was a single-center with a double-blind, randomized parallel-controlled trial design conducted in the Nasibeh dormitory of Tehran between April 2018 and November 2018. The study protocol was presented to and approved by the Research Ethics Committee of Tehran University of Medical Sciences (TUMS) (no. IR.TUMS.VCR.REC.1396.4670) and was then registered at the Iranian Registry of Clinical Trials (IRCT) Center (IRCT20180311039038N1; 03/25/2018). A consent form was taken before initiation of trial from all eligible participants. Participants were aware of the purpose, procedure, advantages, and disadvantages of study and their legal right to withdraw at any stage of study. Moreover, the CONSORT checklist as the guideline for reporting this study was used (see Appendix S1).

### 2.2. Participants

The research team presented a summary of study objectives and protocol to the students and asked them whether they would like to participate in the study or not. A total of 790 students living in the dormitory voluntarily filled out the PMS diagnosis questionnaire for two consecutive cycles. Five hundred forty-eight of them were identified to have a provisional diagnosis of PMS. They were then asked to fill out the Iranian version of the PSST questionnaire to rate their PMS severity. Individuals meeting the following criteria were considered as having PMDD or a very severe case of PMS: (A) Answered “severe” to at least 1 of the 4 first questions. (B) Answered “moderate” or “severe” to at least 4 of the 14 first questions. (C) Answered “severe” to at least 1 of the 5 last questions. Those meeting the following criteria were considered having moderate-to-severe symptoms: (A) Answered “moderate” or “severe” to at least 1 of the 4 first questions. (B) Answered “moderate” or “severe” to at least 4 of the 14 first questions. (C) Answered “moderate” or “severe” to at least 1 of the 5 last questions. Other affected individuals were considered as having mild PMS. Those participants with moderate-to-severe symptoms were then invited by the research team for the interview to select participants meeting the inclusion/exclusion criteria of the study.

The inclusion criteria were as follows: women aged 15–49 years, with regular menstrual cycles of 25–34 days, and with moderate-to-severe PMS according to the PSST questionnaire. The exclusion criteria were being pregnant, considering or trying for pregnancy during study, lactating, getting married during the period of study (as might cause unforeseen stress), diagnosed or having symptoms of any other physical illness such as thyroid disease or anemia, being treated for any mental illness, using other medications such as hormonal or herbal medicines, consuming alcohol, tobacco, or illicit substances, having medication allergy, and being drug intolerant such as having an incidence of severe side effects. Based on the inclusion and exclusion criteria, eligible participants amongst female students living in the same dormitory were identified.

### 2.3. Sample Size Estimation

G^*∗*^power (version 3.1.9) [36], statistical power and sample size calculator software, was used to compute the required sample size. To achieve a moderate effect size (*d* = 0.5) with static power of 0.8 at a significant level of 0.05, a total of 64 patients were needed in each group.

### 2.4. Randomization

The randomization process was done using a custom-made computer program that randomly assigned participants to one of the two groups with 1 : 1 allocation ratio. All the study participants and the research personnel involved in running the experiment were blind to the subjects' group allocation during the study.

### 2.5. Intervention

A single dose of 400 mg tablet per day was taken by all participants for three consecutive cycles. The intervention group received 400 mg Allium-S tablets, while the control group received placebo tablets. Both tablets were manufactured and provided by Dineh Pharmaceutical Company (Qazvin, Iran). The Allium-S tablets were made from dried garlic powder containing 1.1 mg of allicin, its active ingredient, in one 400 mg garlic tablet. The pharmaceutical company made the placebo tablets from starch powder and placed them next to garlic tablets for a month to acquire garlic odor. Placebo and garlic tablets had the same appearance in odor, shape, texture, color, and size. The tablets were encoded by the pharmaceutical company, and the encryption keys were sent to the research team by mail after the completion of the intervention. This allowed both participants and investigators to be blinded to the type of medication during the study.

### 2.6. Study Procedures

On the first day of their first cycle (i.e., cycle 1), participants started taking the daily tablets for three consecutive cycles (i.e., stopped it at the start of cycle 4). After beginning the intervention, participants completed the PSST questionnaire at the beginning of the first cycle according to the last pre-intervention cycle (cycle 0) and then for three consecutive cycles (cycles 1, 2, and 3) early in the subsequent cycle. During the study, a research team member was present at the dormitory and verified the use of tablets and their possible complications and checked with the participants about their compliance with treatment.

### 2.7. Measures

Two questionnaires were used in this study: one to identify/diagnose individuals suffering from PMS and the other to rate the severity of PMS. The first questionnaire (i.e., the PMS diagnostic tool) is a self-assessment questionnaire based on the fourth edition of the Diagnostic and Statistical Manual of Mental Disorders (DSM-IV) of the American Psychiatric Association [37]. It is a questionnaire with a prospective daily rating of symptoms consisting of 11 questions related to (1) depressed mood or dysphoria, (2) anxiety or tension, (3) affective lability, (4) irritability, (5) decreased interest in usual activities, (6) Difficulties in concentrating, (7) marked lack of energy, (8) marked change in appetite, overeating, or food cravings, (9) hypersomnia or insomnia, (10) feeling overwhelmed, and (11) other physical symptoms (such as breast tenderness and bloating). The first four questions are related to affective symptoms. To have a positive PMS diagnosis, multiple criteria need to be met for at least two consecutive symptomatic menstrual cycles. First, it requires a positive response to at least 5 questions, one of them related to the affective symptoms. Then, three criteria must be met: (A) symptoms must occur during the week before menses and remit a few days after onset of menses; (B) symptoms must interfere with work, school, relationships with family, social life, and household responsibilities; and (C) symptoms must not merely be an exacerbation of another disorder. In summary, for at least two consecutive symptomatic menstrual cycles, a person having 5 positive responses and meeting the criteria A, B, and C is identified as the person with PMS.

As the former questionnaire does not measure the severity of PMS, the Iranian version of the premenstrual symptoms screening tools (PSST) questionnaire was used for this purpose [38]. This retrospective self-administrated questionnaire asks participants to rate the listed symptoms/experience if those start before menstruation and stop within the first few days of bleeding. The 4-point Likert-type scale of “not at all,” “mild,” “moderate,” or “severe” was used to rate each question scored from 0 to 3, respectively. The questionnaire consists of 19 questions organized in two sections; the first section assesses the individual's symptoms and impairment during PMS using 14 questions related to physical, behavioral, and mood symptoms. The listed symptoms include depressed mood (hopelessness), anxiety (tension), tearfulness (increased sensitivity to rejection), anger (irritability), decreased interest in work activities, decreased interest in home activities, decreased interest in social activities, difficulty concentrating, fatigue (lack of energy), overeating (food craving), insomnia, hypersomnia, feeling overwhelmed or out of control, and physical symptoms (headaches, muscle aches, chest pain, and flatulence).

The second section consists of 5 questions related to the effects of these symptoms on the quality of life. Women were asked “How your symptoms, as listed above, interfered with any of the following five areas: work efficiency, educational activities, social life, relationships with family, and household responsibilities?” Participants filled out this questionnaire at the beginning of their menstruation, according to symptoms in the previous cycle. The premenstrual disorders criteria listed in this questionnaire are consistent with those of DSM-IV and DSM-V for PMDD [39]. The Iranian version of the PSST was the primary outcome measure of this study. It was also used in identifying eligible participants (i.e., those with moderate-to-severe PMS [40]).

### 2.8. Statistical Analysis

In this study, all analysis was performed by the intent-to-treat (ITT) principle. The intervention and control groups were compared on demographic variables. Qualitative variables were expressed in percentage (%) and analyzed by chi-square test, while quantitative data were represented as mean ± SD and analyzed by independent samples *t*-test. The severity of symptoms was compared between the two groups at the pre-intervention cycle (cycle 0) and the three consecutive cycles after intervention (cycles 1, 2, and 3). The repeated measures ANOVA test was used to compare mean changes within and between two groups in pre-post intervention periods. The Bonferroni post hoc test was used for multiple comparisons. Moreover, a Chi-square test was used to compare potential side effects between groups. Statistical analysis was performed using SPSS 18 software and the statistical significance level was set at the level of *P* < 0.05.

## 3. Results

### 3.1. Study Participants

**Figure 1:**
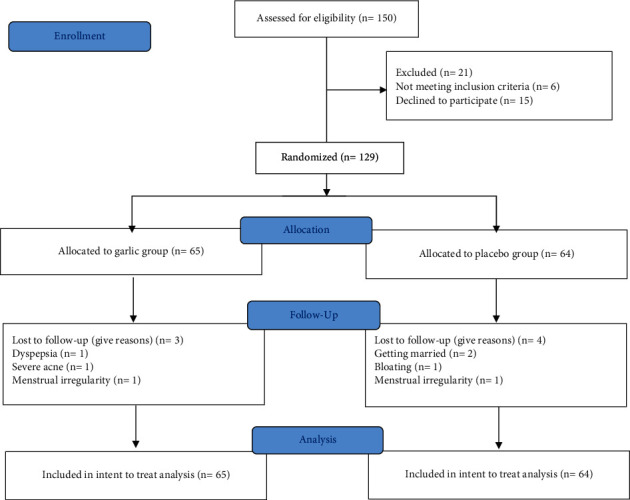
Flow diagram of the study.

21 out of 150 eligible patients declined to participate or did not meet included criteria. From 129 students taking part in the study, 65 women were randomized to the garlic group and 64 to the placebo group. During the investigation, seven persons did not complete the study, whose dropout rates were 3 (4.61%) in garlic and 4 (6.25%) in placebo group (Figure 1). All the 122 participants who completed the study took all their tablets during 3 cycles as instructed.

### 3.2. Demographics

There were no significant differences between the two groups regarding the parameters mentioned in Table 1 (age, body mass index (BMI), duration of the cycle, duration of menstruation, marital status, educational field, and the province of residence), and they were eligible for parallel comparison.

### 3.3. Study Outcomes

As shown in Table 2, there was no significant difference in the parameters of premenstrual symptoms scores before the intervention between the two groups. However, there was a significant difference (*P* < 0.001) after the intervention in the mean changes between the two groups. At the end of the intervention in the garlic group, 59 people (90.76%) were in the mild or disease-free class. That number was 23 (35.93%) in the placebo group.

After intervention for three consecutive cycles, the total score of premenstrual symptoms in the two groups significantly reduced; in the garlic group from 34.09 ± 7.31 to 11.21 ± 7.17 (mean changes: 22.88, 95% CI 20.72–25.03; *P* < 0.001); in the placebo group from 33.35 ± 7.96 to 24.28 ± 7.22 (mean changes: 9.07, 95% CI 7.68–10.46; *P* < 0.001). The difference between the mean changes in each of the three cycles after the intervention was significant (*P* < 0.001). The mean difference was 6.05, 95% CI 3.26–8.84 at cycle 1; 9.85, 95% CI 7.17–12.53 at cycle 2; and 13.07, 95% CI 10.55–15.57 at the end of the intervention. The results of this analysis showed that the main effect of treatment on reducing the mean total score is significant; this reduction was significantly greater in the treatment group than in the placebo group (*P*-value <0.001; *F* (1, 127) = 35.121; partial eta squared = 0.217). Also, the results of Bonferroni post hoc test showed a significant difference between each pair of replicates in both groups (Table 3).

### 3.4. Clinical Complications and Adverse Effects

Nine adverse effects were observed over the intervention. The difference between the garlic and placebo in the frequency of some adverse effects was significant (Table 4). Patients in the garlic group experienced symptoms like acne, itching, and flushing more than placebo, while spotting and bloating occurred more in the control group. There was no considerable difference in other symptoms between groups.

## 4. Discussion

The results of this study highlight the effects of garlic on reducing premenstrual symptoms. Participants in the study had a moderate-to-severe degree of PMS symptoms before the intervention and following three consecutive menstrual cycle interventions, a significant decrease in mean symptom scores was noted in the garlic group compared with placebo.

Although the pathophysiology of premenstrual disorders is not yet fully understood, the behavioral symptoms are thought to be due to the altered stimulatory impact of progesterone and estradiol on dopamine levels in the brain [41]. Evidence suggests that serotonin is involved in the pathophysiology of PMS, particularly in the prevalence of mood and behavioral symptoms [12, 42]. Clinical evidence has verified that premenstrual symptoms are significantly attenuated through serotonergic neurotransmission enhancer drugs (e.g., serotonin reuptake inhibitors) [42, 43].

Despite their proven efficacy, side effects of SSRIs include fatigue, low mood, sleep disturbances, nausea, headache, reduced libido, and difficulty achieving orgasm. Some of these symptoms may lead to treatment discontinuation [44]. Using unopposed estrogen may increase the risk of endometrial cancer and providing endometrial protection with progestogen may induce premenstrual symptoms [45]. Besides, there is a risk of thromboembolic effects in drospirenone-containing OCs [46]. GnRH agonist is usually reserved for severe cases and when treatment has been resistant to the use of SSRIs. The resulting hypoestrogenic state leads to adverse effects such as vaginitis, osteoporosis, and vasomotor symptoms [47]. Risks of bilateral salpingo-oophorectomy, including the need for postsurgical estrogen replacement, should be considered [48]. Data concerning the therapeutic effects of VAC are promising; however, they are still controversial [49]. So, synthetic drugs are not administered to treat premenstrual disorders because of the side effects except in severe cases.

Based on the findings of this study, garlic can be practical for the treatment of premenstrual symptoms. Globally, the beneficial effects of garlic in the prevention and treatment of diseases have been proven in previous studies. Immunoregulation and modulation of secretion of cytokines by *Allium sativum* may be the mechanism of action of many of its therapeutic effects (antidiabetic, antihypertensive, and hypolipidemic) for metabolic syndrome [31, 50, 51]. Compelling evidence supports the ability of aged garlic extract (AGE) to protect against oxidant-induced diseases, that is, reduced risk of cardiovascular disease, stroke, cancer, and aging, including oxidant-mediated brain cell damage in neurodegenerative disorders, especially Alzheimer's disease (AD) [52–54]. The findings of several studies suggest that garlic intake may lead to inhibition of *β*-amyloid protein (A*β*) aggregation in the human brain [55–58].

The use of a diet supplement containing garlic and black sesame in ovariectomized rats has shown significant antidepressant-like activity [35]. Garlic also reduces anxiety and depression behaviors in diabetic rats, possibly by reducing brain oxidative stress [32]. AGE may improve memory by affecting cholinergic, glutamatergic, and GABAergic systems concerning cognitive impairment in A*β*-induced rats [34, 59]. Evidence for the antidepressant-like activity of garlic extract in mice has been found through inhibition of monoaminoxidase-A (MAO-A) and monoaminoxidase-B (MAO-B) and involvement of adrenergic, dopaminergic, serotoninergic, and GABAergic systems [33]. Evidence also shows that administration of garlic in rats with increased brain serotonin (5-hydroxytryptamine) levels improves cognitive performance [60]. Collectively, these beneficial effects of garlic on improving cognitive and mood symptoms confirm that part of the impact of garlic in our study may be through boosted levels of serotonin and dopamine in the brain.

Besides the beneficial effects of garlic, it is usually accompanied by some mild side effects. In this study, participants in the garlic group announced some mild complaints, such as itching, flushing, and acne. Some studies showed that garlic could induce allergic reactions and irritant dermatitis [61]. Also, heartburn, gastrointestinal irritation, and nausea can be potential side effects of garlic consumption [62].

In addition to the randomized, placebo-controlled, and double-blind design, this study has several strengths. The participants received daily treatment for three consecutive cycles, while in most studies related to premenstrual symptoms, the intervention has been only in the luteal phase. This study had a suitable distribution and participants were present from 26 of the 31 provinces of the country. We could not find any investigation on the effect of garlic on PMS to compare with our study results. In this study, garlic effectively reduced PMS symptoms with no side effects. The main effect of comparing the two types of intervention was significant and large. However, as with some other PMS treatment studies, the placebo effect was significant [63]. It is likely that participants hoped for disease treatment and the expectation of relief of premenstrual symptom severity could reduce their mental stress and alleviate symptoms.

There are some limitations to this study. The voluntary basis of this study opens it to a systematic difference between persons who choose to participate. The study also has the limitation of all self-assessment questionnaires including social desirability bias. The generalizability of the results might be a potential problem as it has been done in only one dormitory. Also, the short duration of study highlights a need for further studies with a longer follow-up period to assess the long-lasting effect of the garlic. Further studies could be done in multiple centers and offer better generalizability by removing some of the mentioned limitations. Future studies are needed to evaluate the effects of garlic on the neurotransmitters involved in premenstrual symptoms.

## 5. Conclusion

Our findings revealed benefits of garlic for treating premenstrual symptoms without severe side effects. Since many women nowadays tend to use herbal remedies to prevent and treat premenstrual symptoms because of the side effects of synthetic drugs, garlic can be considered as a complementary medicine to improve premenstrual symptoms.

## Figures and Tables

**Table 1:** Demographic data of the participants in the two groups of the study.

Parameter	Garlic group (*n* = 65)	Placebo group (*n* = 64)	*P*-value
Age (year); mean ± SD	21.5 ± 1.4	20.9 ± 1.3	0.170
Weight (kg); mean ± SD	58.5 ± 7.9	58.6 ± 8.1	0.934
Height (cm); mean ± SD	164.1 ± 4.5	162.9 ± 5.0	0.168
BMI (kg/m^2^); mean ± SD	21.7 ± 2.8	22.0 ± 2.6	0.483
Duration of menstruation (day); mean ± SD	6.2 ± 1.1	6.4 ± 1.0	0.329
Duration of cycle (day); mean ± SD	29.6 ± 2.1	30.1 ± 1.8	0.205
Marital status			
Single; number (%)	54 (83.1%)	44 (69.0%)	0.057
Married; number (%)	11 (16.9%)	20 (31.0%)	
Educational field			
Physical education; number (%)	6 (9.2%)	2 (3.1%)	0.151
Nonphysical education; number (%)	59 (90.8%)	62 (96.9%)	
Province; (total: 31 provinces)			
Number (%)	21 (68%)	20 (65%)	0.160

Resulted from independent *t*-test for quantitative and chi-square test for categorical variables.

**Table 2:** Premenstrual symptoms scores in placebo and garlic groups before (cycle 0) and after intervention (cycles 1, 2, and 3).

Symptoms		Groups		Mean difference between groups (95% CI)	*P*−value
		Placebo (*n* = 64) mean ± SD	Garlic (*n* = 65) mean ± SD		
*Mood symptoms*	Cycle 0 Cycle 1 Cycle 2 Cycle 3	7.43 ± 2.18 6.28 ± 2.21 5.76 ± 2.02 5.18 ± 1.85	7.56 ± 2.09 4.95 ± 1.92 3.67 ± 2.03 2.33 ± 1.92	−0.13 (−0.08–0.61) 1.32 (0.60–2.04) 2.08 (1.37–2.79) 2.84 (2.18–3.50)	0.727 <0.001 <0.001 <0.001

*Behavioral symptoms*	Cycle 0 Cycle 1 Cycle 2 Cycle 3	13.29 ± 4.38 11.73 ± 3.82 10.50 ± 3.48 9.57 ± 3.27	13.46 ± 3.87 9.53 ± 4.16 7.24 ± 4.04 5.07 ± 3.58	−0.16 (−1.60–1.27) 2.19 (0.80–3.58) 3.25 (1.93–4.57) 4.50 (3.30–5.69)	0.822 0.002 <0.001 <0.001

*Physical symptoms*	Cycle 0 Cycle 1 Cycle 2 Cycle 3	5.64 ± 2.52 5.10 ± 2.74 4.54 ± 2.63 4.06 ± 2.43	5.50 ± 3.01 3.81 ± 2.24 2.72 ± 2.24 1.93 ± 1.86	0.13 (−0.83–1.10) 1.29 (0.43–2.16) 1.82 (0.97–2.67) 2.12 (1.36–2.88)	0.787 0.004 <0.001 <0.001

*Interfering symptoms*	Cycle 0 Cycle 1 Cycle 2 Cycle 3	6.98 ± 2.34 6.26 ± 2.53 5.87 ± 2.32 5.43 ± 2.18	7.55 ± 2.03 5.03 ± 2.41 3.18 ± 2.05 1.86 ± 1.77	−0.56 (−1.33–0.19) 1.23 (0.37–2.09) 2.69 (1.92–3.45) 3.57 (2.88–4.26)	0.143 0.005 <0.001 <0.001

*Total symptoms*	Cycle 0 Cycle 1 Cycle 2 Cycle 3	33.35 ± 7.96 29.39 ± 8.33 26.68 ± 7.58 24.28 ± 7.22	34.09 ± 7.31 23.33 ± 7.67 16.83 ± 7.78 11.21 ± 7.17	−0.74 (−3.99–1.93) 6.05 (3.26–8.84) 9.85 (7.17–12.53) 13.07 (10.55–15.57)	0.587 <0.001 <0.001 <0.001

Resulted from repeated measures ANOVA test based on comparing the mean difference between two groups.

**Table 3:** Mean difference changes of scores of the PMS symptoms in the two groups before and after the intervention.

Parameters	Mean difference (95% CI) within placebo group (*n* = 64)	Mean difference (95% CI) within garlic group (*n* = 65)	Mean difference changes (95% CI) between groups	*P*-value	Partial eta squared	*F* (1,127)
Mood symptoms	2.25 (1.77–2.72)	5.23 (4.56–5.89)	2.98 (2.16–3.79)	<0.001	0.177	27.30
Behavioral symptoms	3.71 (2.79–4.64)	8.38 (7.47–9.29)	4.66 (3.38–5.95)	<0.001	0.120	17.28
Physical symptoms	1.57 (1.06–2.08)	3.56 (2.90–4.23)	1.99 (1.16–2.82)	<0.001	0.086	11.96
Interfering symptoms	1.54 (1.08–2.01)	5.69 (5.07–6.31)	4.14 (3.37–4.91)	<0.001	0.173	26.49
Total symptoms	9.07 (7.68–10.46)	22.88 (20.72–25.03)	13.78 (11.23–16.33)	<0.001	0.217	35.121

Resulted from repeated measures ANOVA test and Bonferroni post hoc test.

**Table 4:** Clinical complications and adverse effects were reported as number per group.

Adverse effects	Garlic (*n* = 65)	Placebo (*n* = 64)	*P*-value
Acne	0	7	0.007
Flushing	1	9	0.009
Itching	0	6	0.013
Bloating	11	0	<0.001
Spotting	5	0	0.022
Dyspepsia	1	5	0.098
Nausea	2	8	0.051
Dizziness	8	8	0.974
Hypermenorrhea	0	1	0.319

Resulted from chi-square test.

## Data Availability

The data used to support the findings of this study are available from the corresponding author upon request.

## Abbreviations

AGE: Aged garlic extract
AD: Alzheimer's disease
A*β*: *β*-Amyloid protein
BMI: Body mass index
CAM: Complementary and alternative medicine
CBT: Cognitive-behavior therapy
DSM-IV: Diagnostic and Statistical Manual of Mental Disorders, 4th edition
DSM-V: Diagnostic and Statistical Manual of Mental Disorders, 5th edition
GABA: Gamma-aminobutyric acid
GnRH: Gonadotropin-releasing hormone
IRCT: Iranian Registry of Clinical Trials
ISPMD: International Society for the Study of Premenstrual Disorders
MAO-A: Monoaminoxidase-A
MAO-B: Monoaminoxidase-B
OCs: Oral contraceptives
PSST: Premenstrual symptoms screening tools
PMD: Premenstrual disorder
PMDD: Premenstrual dysphoric disorder
PMS: Premenstrual syndrome
SSRIs: Selective serotonin reuptake inhibitors
TUMS: Tehran University of Medical Sciences
US FDA: United States Food and Drug Administration
VAC: Vitex agnus castus.

## Supplementary Materials

Supplementary Materials CONSORT 2010 checklist of information to include when reporting a randomized trial.
